# Memantine Modulates Oxidative Stress in the Rat Brain following Experimental Autoimmune Encephalomyelitis

**DOI:** 10.3390/ijms222111330

**Published:** 2021-10-20

**Authors:** Beata Dąbrowska-Bouta, Lidia Strużyńska, Marta Sidoryk-Węgrzynowicz, Grzegorz Sulkowski

**Affiliations:** Laboratory of Pathoneurochemistry, Department of Neurochemistry, Mossakowski Medical Research Institute, Polish Academy of Sciences, 5 Pawińskiego Str., 02-106 Warsaw, Poland; bbouta@imdik.pan.pl (B.D.-B.); lidkas@imdik.pan.pl (L.S.); msidoryk@imdik.pan.pl (M.S.-W.)

**Keywords:** EAE, glutamate receptor antagonist, excitotoxicity, oxidative stress, -SH groups, superoxide dismutase

## Abstract

Experimental autoimmune encephalomyelitis (EAE) is an animal model most commonly used in research on the pathomechanisms of multiple sclerosis (MS). The inflammatory processes, glutamate excitotoxicity, and oxidative stress have been proposed as determinants accompanying demyelination and neuronal degeneration during the course of MS/EAE. The aim of the current study was to characterize the role of NMDA receptors in the induction of oxidative stress during the course of EAE. The effect of memantine, the uncompetitive NMDA receptor antagonist, on modulation of neurological deficits and oxidative stress in EAE rats was analyzed using several experimental approaches. We demonstrated that the expression of antioxidative enzymes (superoxide dismutases SOD1 and SOD2) were elevated in EAE rat brains. Under the same experimental conditions, we observed alterations in oxidative stress markers such as increased levels of malondialdehyde (MDA) and decreased levels of sulfhydryl (-SH) groups, both protein and non-protein (indicating protein damage), and a decline in reduced glutathione. Importantly, pharmacological inhibition of ionotropic NMDA glutamate receptors by their antagonist memantine improved the physical activity of EAE rats, alleviated neurological deficits such as paralysis of tail and hind limbs, and modulated oxidative stress parameters (MDA, -SH groups, SOD’s). Furthermore, the current therapy aiming to suppress NMDAR-induced oxidative stress was partially effective when NMDAR’s antagonist was administered at an early (asymptomatic) stage of EAE.

## 1. Introduction

Experimental autoimmune encephalomyelitis (EAE) is the most popular and well characterized animal model of multiple sclerosis (MS). MS is an immune-mediated demyelinating disease of the central nervous system (CNS) with inflammatory and neurodegenerative components that often affects young adults between the ages of 20 and 40, more frequently female than male [1]. The characteristic features of the disease are demyelinating areas in the white and grey matter of the spinal cord and brain. The etiology of MS is still not fully understood, although the role of both genetic and environmental factors has been demonstrated [1]. In demyelinating lesions, the presence of lymphocytes, macrophages and activated microglia has been observed in the proximity of the perivascular area [2,3], suggesting that these types of cells are involved in the process of demyelination [4]. Macrophages and reactive microglia activate the complement pathway, produce pro-inflammatory cytokines, release excitatory amino acids, and generate free radicals [2]. All of these factors can damage myelin and oligodendrocytes and, consequently, disrupt neurotransmission or induce injury and death of neurons. Inflammation, glutamate excitotoxicity and oxidative stress have all been proposed as the most important determinants associated with demyelination and degeneration of neurons during the course of MS [2,5,6].

Additionally, in the EAE rat brain, glutamate-induced cell death and activation of glial cells (microglia and astrocytes) has been reported, which consequently leads to the production and release of inflammatory mediators, such as cytokines and chemokines, and oxygen free radicals [7,8,9].

Glutamate is the main excitatory neurotransmitter in the mammalian brain and plays an important role in both physiological and pathological mechanisms operating in the CNS. The extracellular level of glutamate must be tightly controlled because an excess of this neurotransmitter leads to excitotoxic cell death [10]. In MS, excitotoxicity is caused by different mechanisms, but the ultimate consequence is neuronal damage due to overstimulation of glutamate receptors (GluRs), especially of NMDA subtype receptors [11]. Correlations between altered glutamate homeostasis, cell death, axonal damage, and disturbances in glutamatergic neurotransmission have been identified during both MS and EAE [2,12,13]. The exact mechanisms of glutamate-mediated disturbances are still not fully understood, but the overstimulation of glutamate receptors has been shown to be a main cause of the excessive production of reactive oxygen and nitrogen species (ROS/RNS) and the resulting oxidative stress (OS) [14]. It is widely accepted that acute glutamate-induced neuronal degeneration is mainly mediated by NMDA receptors, the activation of which leads to a massive influx of extracellular Ca^2+^ into the cells followed by an increase in intracellular Ca^2+^ concentration to pathological levels [10]. Increased intracellular Ca^2+^ levels may further lead to a series of downstream neurotoxic cascades, resulting in the increased formation of ROS and activation of both caspase-dependent and caspase-independent cell death, in which mitochondria play a key role [15]. Ca^2+^ overload depolarizes the mitochondrial membrane and initiates OS mediated by the mitochondrial pathway [16]. Overproduction of ROS causes deleterious effects on proteins, lipids, and nucleic acids, leading to the disruption of cellular functions [17]. High intracellular Ca^2+^ levels also activate a number of Ca^2+^-dependent enzymes such as proteases, phospholipases, kinases, nitric oxide synthase (NOS), and endonucleases, which mediate proteolysis, free-radical production, or lipid peroxidation, thereby enhancing OS and subsequent oligodendrocyte and neuronal death. Ca^2+^-related stimulation of mitogen-activated protein kinase p38 (MAPK p38) activates transcription factors that modify the nucleus and cause neuronal injury and apoptosis [8,10,18,19,20]. Several studies also suggest that overstimulation of GluRs may contribute to the pathogenesis of MS/EAE by altering the integrity of the blood–brain barrier (BBB). The mechanisms of GluRs-mediated changes in neurovascular integrity are unclear but have been shown to involve vasoactive molecules such as nitric oxide (NO) and superoxide radical (O_2_˙) that can combine to form damaging levels of peroxynitrite (ONOO^−^) [21,22]. Therefore, it appears that glutamate has the potential to mediate both myelin and BBB breakdown in MS/EAE via the action of free radicals and up-regulation of various enzymes [23].

Under physiological conditions, ROS/RNS are neutralized by enzymatic (superoxide dismutases-SODs, catalase, and peroxidase) and non-enzymatic (glutathione, uric acid, and ascorbic acid) antioxidant defense systems. Under pathological conditions, elevated levels of ROS/RNS, which are produced as a result of inflammatory processes, mitochondrial respiratory chain dysfunction, or overstimulation of glutamate NMDA receptors, induce a tissue response that includes upregulation of various scavenger molecules, such as cytosolic SOD1 and mitochondrial SOD2 (the SOD family enzymes most commonly expressed in the CNS). Superoxide anion is neutralized by SOD, which converts the radical to the less toxic hydrogen peroxide, which is further neutralized by the antioxidant enzyme catalase and via the glutathione peroxidase pathway. The level of SODs has been reported to increase in astrocytes and macrophages of the brain of MS patients [5,24,25].

When ROS are not counterbalanced by cellular antioxidative defense systems, ROS metabolites cause OS, leading to protein and lipid peroxidation and DNA alkylation, all of which were observed in MS patients [26]. The induction of lipid peroxidation may be a major factor in free radical-mediated CNS damage. It is a complex process involving the interaction of oxygen-derived free radicals with polyunsaturated fatty acids, resulting in the formation of a variety of highly active electrophilic aldehydes with harmful potential [27].

In the current study, we assessed the contribution of NMDA glutamate receptors to oxidative/nitrosative stress during EAE. We investigated whether inhibition of NMDA receptors by their antagonist, memantine, affects markers of oxidative stress in the brain of rats subjected to EAE. An experimental approach included an analysis of the changes in the parameters indicating the oxidation of proteins (the level of protein sulfhydryl groups -SH) and lipids (the level of malondialdehyde, MDA). Furthermore, we evaluated the relevant antioxidative mechanisms focusing on the estimation of the enzymatic (SOD1 and SOD2) and non-enzymatic (glutathione-related non-protein -SH group) status during the different phases of the disease.

## 2. Results

### 2.1. The Effect of Memantine on the Course of EAE

The first symptom during the course of EAE is a change in body weight. In all experimental groups, except the control group, the rats achieved the highest body weight at about 8 d.p.i. At this time, body weight was in the same range for both the EAE untreated and EAE drug treated groups. From 8 d.p.i. to 14 d.p.i., EAE rats underwent a progressive 20–30% weight loss compared to their body weight at the beginning of the experiment, which corresponded to the acute phase of the disease and maximal neurological deficits. At 25 d.p.i., the mean body weight returned to the value observed at the beginning of the experiment. In the groups of EAE rats treated with NMDAR antagonist memantine, weight loss was significantly lower (by about 15%) relative to EAE rats (untreated) (Table 1).

**Figure 1:**
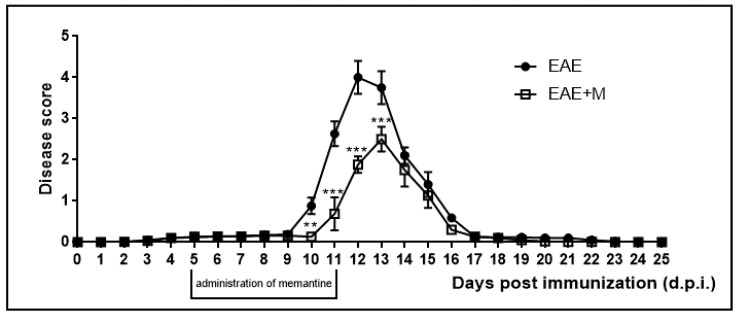
Scores of the neurological symptoms in experimental autoimmune encephalomyelitis (EAE) and memantine-treated EAE rats in different phases of the disease between 0 and 25 days post immunization (d.p.i.). Memantine was administered at a dose of 60 mg/kg b.w./day from 5 to 11 d.p.i. The values indicated neurological scores ± S.D. Results are combined data from 8 animals in each group. ** *p* < 0.01 *** *p* < 0.001 vs. untreated EAE rats (Student’s *t*-test).

The neurological deficits observed during the course of EAE were classified daily. Neurological symptoms of the disease included developmental paralysis of tail and hind limbs and reduced physical activity of experimental rats. The neurological symptoms of EAE started to develop at 10–11 d.p.i. and peaked at 12–13 d.p.i. At 14 d.p.i., rats achieved partial recovery from neurological symptoms and full recovery was observed at 17 d.p.i. We did not observe any further neurological symptoms of the disease until the end of the experiment at 25 d.p.i. (Figure 1). Clinical parameters and the effects of memantine are presented in Table 2 and have been described in detail in our previous publications [28,29,30]. In the current study we observed a reduction in the severity and duration of neurological deficits after the administration of memantine. All rats in the memantine-treated EAE group showed better physiological conditions than the untreated EAE rats. Notably, the duration of the acute phase of the disease was also shortened by 1–2 days in the memantine-treated EAE group compared to the EAE rats. The lethality observed in the EAE rats after the administration of memantine, although not significant, was noticeable but was found to be lower than that of the EAE untreated rats.

### 2.2. The Effect of NMDA Receptor Antagonists on the Level of Oxidative Stress Markers

**Figure 2:**
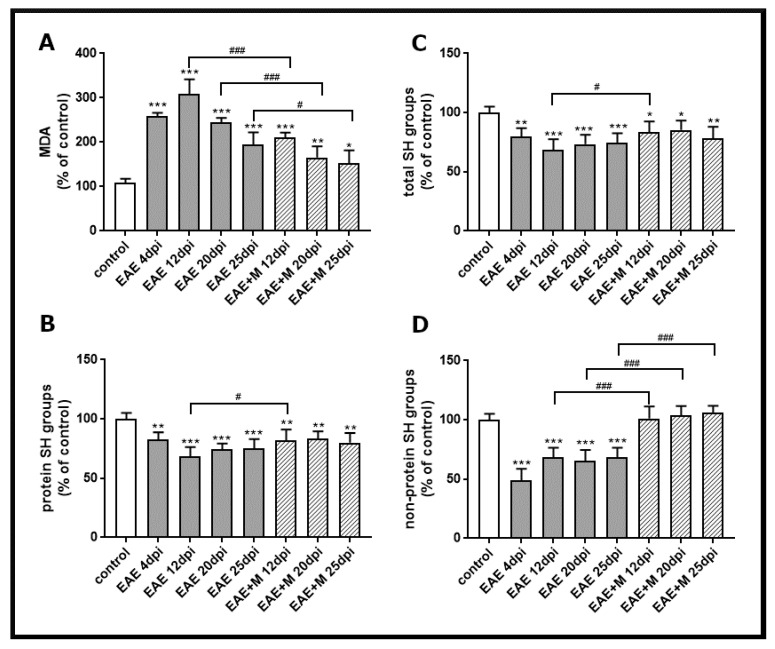
Thiobarbituric acid reactive substances (TBARS) content expressed as the level of malondialdehyde (MDA), a product of membrane lipid peroxidation (**A**), the level of total SH groups (**B**), protein SH groups (**C**), and non-protein SH groups (**D**) in brain homogenate obtained from the control, experimental autoimmune encephalomyelitis (EAE) rats and memantine-treated EAE rats in different phases of the disease. The results are the means from *n* = 4 animals and are expressed as percentages of the control. * *p* < 0.05, ** *p* < 0.01, *** *p* < 0.001 significantly different vs. control rats. ^#^ *p* < 0.05, ^###^ *p* < 0.01 significantly different vs. untreated EAE rats (one-way ANOVA with post hoc Bonferroni’s test).

To investigate the involvement of the NMDA receptor antagonist on the processes related to oxidative stress, we analyzed lipid peroxidation by measuring the concentration of a small end-product of oxidized fatty acid degradation, malondialdehyde (MDA). The obtained results showed that the level of MDA significantly increased between 4 and 25 d.p.i. in all experimental groups by about 40–60% compared to the control (Figure 2A). After administration of memantine, MDA levels decreased by about 50–100% between 12 and 25 d.p.i. relative to untreated EAE rats (Figure 2A).

The levels of total -SH groups (Figure 2C), protein -SH groups (Figure 2B), and non-protein -SH groups (Figure 2D) in brain homogenates obtained from untreated EAE rats decreased by about 20%, 30%, and 60%, respectively, compared to the control at 4 d.p.i., and these changes were stable until 25 d.p.i. (Figure 2B–D). The administration of memantine to EAE rats significantly prevented the decrease in non-protein-SH groups (Figure 2D) between 12 and 25 d.p.i. Moreover, it increased the levels of total protein (by about 20%) and protein–SH groups (by about 30%) at 12 d.p.i. (Figure 2B,C).

### 2.3. Memantine-Induced Changes in the Expression of SOD Enzymes

To investigate the expression of mRNA coding for of SOD1 and SOD2 during the course of EAE and after treatment with memantine, we performed a qPCR test. Western blot was used to estimate immunoreactivity of SOD1 and SOD2 proteins in brain homogenate obtained from control rats, rats with EAE and memantine-treated rats. A strong positive immunoreaction was observed in a single band near 18 kDa and 25 kDa for SOD1 and SOD2, respectively.

**Figure 3:**
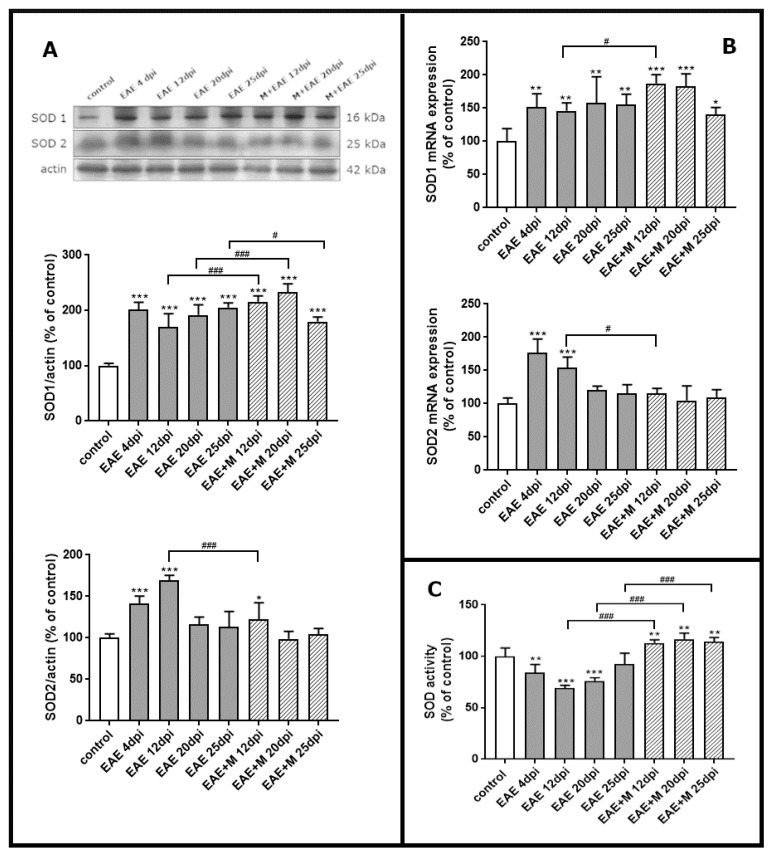
The expression of superoxide dismutases SOD1 and SOD2 in the brain of control and experimental autoimmune encephalomyelitis (EAE) rats at different times post-immunization and after therapeutic treatment with memantine. Representative immunoblots and bars showing the relative levels of enzymatic protein (**A**), bars showing the expression of enzymatic mRNA (**B**), and bars showing the total activity of SODs enzymes (**C**). The results are the means from *n* = 4 animals in each group and are expressed as a percentage of control. * *p* < 0.05; ** *p* < 0.01; *** *p* < 0.001 significantly different vs. control rats; ^#^ *p* < 0.05, ^###^ *p* < 0.01 significantly different vs. EAE rats not subjected to therapy. (one-way ANOVA followed by Bonferroni’s multiple comparison post-test).

The results revealed a statistically significant increase of around 50% in SOD1 mRNA in EAE rats between 4 and 20 d.p.i. compared to controls. At the end of the experiment (25 d.p.i.), the level of SOD1 mRNA was still above the control value (Figure 3B). After administration of memantine, it was found that EAE-developing animals still exhibited higher SOD1 mRNA expression levels than untreated EAE rats (Figure 3B), while significant upregulation of the enzyme by memantine (by about 20%) was only observed at 12 d.p.i. Identified changes in mRNA expression corresponded to the alterations observed in protein levels. The immunoreactivity of the SOD1 protein in EAE rats remained elevated above control values within the range of 90–110% at 4–20 d.p.i. (Figure 3A). Administration of memantine resulted in the increased expression of SOD1 protein by 10–20% relative to untreated EAE rats at 12–20 d.p.i. The memantine-treated rat model exhibited a 15% decrease in SOD1 protein compared to EAE untreated animals at 25 d.p.i. (Figure 3A).

Alterations in the SOD2 mRNA expressions and protein levels were found to be similar to those observed in SOD1 expression only between 4 and 12 d.p.i., reaching an elevation of about 70–80% compared to the control value (Figure 3D). In agreement with this observation, an elevated expression of SOD2 protein was also observed at 4–12 d.p.i., reaching 50–70% of control (Figure 3A). Interestingly, memantine administration significantly reduced both mRNA and SOD2 protein levels to control values at 12 d.p.i. (Figure 3A,B).

### 2.4. Memantine-Dependent Changes in SOD’s Activity

We next investigated whether memantine administration affected specific enzyme activity. As shown in Figure 3C, total SOD activity markedly decreased in EAE rats compared to the control by about 20%, 40%, and 30% at 4, 12, and 20 d.p.i., respectively. In the group of animals treated with memantine, we observed a statistically significant increase in SOD activity of about 60%, 40%, and 20% compared to untreated EAE animals at 12, 20, and 25 d.p.i, respectively, and by about 10% relative to the control group (Figure 3C).

## 3. Discussion

In the current study, we analyzed the effect of the uncompetitive NMDA receptor antagonist, memantine, on the selective parameters of oxidative stress and neurological deficits in EAE rats. We demonstrated that administration of memantine: (i) significantly decreases the level of MDA relative to untreated EAE rats; (ii) significantly prevents the decrease in non-protein-SH groups and slightly increases the levels of total protein and protein–SH groups at a peak of the disease; (iii) increases expression of SOD1 protein relative to untreated EAE rats; (iv) significantly increases SOD activity compared to untreated EAE animals in all experimental groups; and (v) improves the physiological condition of immunized rats and partially ameliorates clinical symptoms.

Memantine, like other agents that specifically block the pathological stimulation of iGluRs (mainly of the NMDA class), might be expected to restore physiological function of synaptic nerve transmission and produce positive disease-modifying effects. Memantine is a weak, uncompetitive antagonist of the NMDA receptor that has been approved by the European Union and the U.S. Food and Drug Administration (FDA) for the treatment of dementia and Alzheimer’s disease. In contrast to other NMDAR channel blockers such as dizocilpine, ketamine, and phencyclidine, which also inhibit NMDA receptors in a noncompetitive manner, memantine does not exhibit serious side effects [31]. Due to its uncompetitive antagonism and relatively fast off-rate, memantine blocks excessive NMDAR activation but allows for low (physiological) levels of NMDAR activity seen during normal neurotransmission. This may be due to the fact that it more effectively inhibits the extra-synaptic than the synaptic subpopulation of the NMDARs. Importantly, memantine binds at the ‘intracellular’ Mg^2+^ site in the channel pore and displays differential affinity for specific and non-specific binding sites of the NMDAR. These molecular interactions confer upon memantine favorable kinetic properties that contribute to the clinical tolerability of the drug, as well as its neuroprotective profile [32,33].

The potential use of NMDAR antagonists as neuroprotective agents has been established in preclinical studies [32]. Memantine has been found to reduce the NMDA-induced neuronal loss in culture [34] and to be effective in relieving symptoms of MS/EAE [28,29,30,35]. In our previous [28,29,30] and present studies, we investigated the neuroprotective effects of memantine in a rat model of MS. The method of administration and optimal therapeutic doses of the drug were selected based on the previously published data [12,35] and our own experience. The drug was administered starting on days 7 to 11 post-immunization, i.e., in an asymptomatic phase of the disease. We observed that memantine effectively reduced the development and duration of neurological deficits and modified all assessed parameters of the disease. The clinical status of treated rats was significantly improved and the severity of their developed neurological deficits was reduced compared to untreated EAE rats. After therapy with memantine, the disease score decreased to 2.2–2.6, while in untreated EAE rats, it was still 4.5. Additionally, the duration of the disease was reduced by about 2–3 days, whereas the inductive phase was prolonged by about 2 days relative to untreated animals.

The contribution of oxidative stress to the mechanisms operating during immune-mediated inflammatory disease has been previously demonstrated [2,5,6,23,27]. Tissue damage by ROS/RNS was an important cytotoxic mechanism of myeloid immune cells [25,36] and the presence of ROS/RNS has consistently been demonstrated in acute and chronic active MS lesions in correlation with the severity of the disease [36]. Within MS brains, active demyelinating lesions, in particular, show signs of ongoing severe OS as demonstrated by the extensive accumulation of oxidized phospholipids, proteins, and DNA, as well as the enhanced expression of antioxidant factors [37]. Our current study indicates that the applied rodent model of EAE replicates key aspects of the contribution of OS to MS/EAE pathology. The results of our study show changes in the level of both OS markers and antioxidant enzymes. The rate of lipid peroxidation (expressed as MDA level) in the brains of all experimental groups was elevated at all stages of the disease, showing a downward trend only at a late stage of EAE (25 d.p.i.), and was consistent with the data showing the high level of lipid peroxidation (LP) at 15 d.p.i. in the CNS of EAE rats [38,39]. In addition, the level of thiol groups, both protein-bound and non-protein-bound, decreased in the brain homogenates of EAE untreated rats from 4 to 25 d.p.i., suggesting the oxidative damage of proteins and a decrease in reduced forms of glutathione, a non-enzymatic antioxidant defense system. The results correspond with those observed in the early stages of EAE in mice, where the decline in GSH of the brain and spinal cord were also noticed [38]. The administration of memantine significantly prevented the oxidative damage of lipids and restored the levels of non-protein-SH groups at all the examined time points, while it significantly increased the concentration of protein-related and total -SH groups only at 12 d.p.i. Parallel to the markers indicating the presence of oxidative damage, we observed the upregulation of the enzymatic antioxidant defense system, represented by SOD enzymes. The expression of SOD1 was noticeably enhanced over the control during the whole course of EAE at both mRNA and protein levels. Interestingly, SOD2 protein and mRNA levels were elevated only in the early (4 d.p.i.) and acute (12 d.p.i.) phases of EAE, but not in the late phase of the disease (20–25 d.p.i.). Although the expression of both enzymes was enhanced, total SOD activity decreased below the control values in the early and late phases of the disease (12–25 d.p.i.), which is consistent with the previously published data [38]. It has been demonstrated that SOD2 plays a critical role in the mitochondrial signaling pathway and is particularly vulnerable to inactivation by ROS and RNS (RNS in particular), which nitrates its tyrosine residues [40]. The appearance of the SOD2 upregulation in our model may represent the compensatory mechanism of cellular systems to the diminished activity. Administration of memantine not only significantly prevents the loss of total SOD activity by reducing NMDA-mediated oxidative stress, but also upregulates the enzymes, particularly SOD1.Świadczy o tym również wzrost aktywności enzymatycznej SOD obserwowany u zwierząt z EAE między 4-20 d.p.i.

Taken together, our results suggest that blocking the NMDAR-mediated overproduction of ROS/RNS partially reduces the oxidative stress-related damage of lipids and proteins by stimulation of the SOD1-relevant antioxidant defense system. Notably, all these changes are accompanied by the improvement of the physiological condition of immunized rats and partial amelioration of EAE clinical symptoms. The current evidence described here highlights the positive effects of memantine that are important from the clinical point of view, suggesting a new therapeutic strategy for MS treatment.

## 4. Materials and Methods

### 4.1. Animal Model

Female Lewis rats weighing approximately 190–200 g were used throughout the study. Females are more often used in the EAE model because they are more susceptible to disease induction than males [41], reflecting the tendency observed in humans [1]. All procedures were carried out in accordance with ethical guidelines for the care and use of laboratory animals and were approved by the IV Local Care of Experimental Animal Committee in Warsaw (61/2009). To induce EAE, we immunized rats subcutaneously in both hind feet with an inoculum containing guinea pig spinal cord homogenate emulsified in Freund’s complete adjuvant containing 5.5 mg/mL *Mycobacterium tuberculosis* H37Ra (Difco, Detroit, MI, USA).

During the experiment, the rats were housed in environmentally controlled conditions and were provided with free access to food and water. Body weight and neurological deficits were determined daily according to the following scale: 0 = no signs of neurological deficits, 1 = flaccid tail, 2 = impairment of fighting reflex and/or loss of muscle tone in hind limbs, 3 = complete paralysis of hind limbs, 4 = paraplegia, and 5 = a moribund state or death [42,43,44]. Sham-immunized rats (the control group) received subcutaneous injections of Freund’s complete adjuvant containing exclusively *M. tuberculosis* (Difco, Detroit, MI, USA).

Glutamate receptor antagonist memantine was administered at a dose of 60 mg/kg b.w./day. Memantine was dissolved in PBS and administered intraperitoneally to the EAE rats once daily for 7 consecutive days, starting from day 5 post immunization (p.i.) until day 11 p.i. [12].

### 4.2. Experimental Groups and Tissue Processing

A total of 64 animals were used in the study. The rats were arranged into 8 groups (one control group and seven experimental groups sacrificed at different phases of EAE with different recovery periods after treatment with memantine): group I, control (healthy); group II, EAE 4 d.p.i.; group III, EAE 12 d.p.i.; group IV, EAE 20 d.p.i.; group V, EAE 25 d.p.i.; group VI, EAE 12 d.p.i. + memantine; group VII, EAE 20 d.p.i. + memantine; and group VIII—EAE 25 d.p.i. + memantine. Each group consisted of 8 animals. During the experiments, rats were monitored until days 4, 12, 20, and 25 after the initial injection of EAE-inducing inoculum.

At the respective time points, four rats from each experimental group were sacrificed for biochemical analysis and four rats were sacrificed for immunoblotting and real time PCR analysis. The determination of SOD activity was performed in fresh brain homogenates stored on ice and assayed the same day. The others’ brains were quickly removed, frozen in liquid nitrogen and stored at −80 °C for further experiments that included extraction of RNA or preparation of tissue homogenates. To obtain homogenates for immunoblots, the forebrains were homogenized in 50 mM phosphate buffer (pH 7.4) containing 10 mM EGTA, 10 mM EDTA, 0.1 mM PMSF, and 100 mM NaCl in the presence of a protease inhibitor cocktail (1 µg/mL leupeptin, 0.1 µg/mL pepstatin and 1 µg/mL aprotinin).

### 4.3. Measurement of Lipid Peroxidation

Lipid peroxidation was measured in the brain homogenates using the thiobarbituric acid (TBAR) test, according to the methods reported by Wilbur and modified by Asakawa and Matsushita [45]. This method determines the concentration of malondialdehyde (MDA), which is the most important end-product of lipid peroxidation. Forebrains were homogenized and suspended in Krebs-Ringer buffer (pH = 4.0). The samples were preincubated with 25 μM Fe^3+^, 800 μMadenosine diphosphate (ADP), and 200 μMascorbate at 30 °C in a water bath. After incubation, 1 mL of 30% TCA, 0.1 mL of 5 M HCl, and 1 mL of 0.75% TBAR were added. The mixture was heated at 100 °C for 15 min in boiling water and centrifuged. The optical density of the supernatant was determined at 535 nm. The molar extinction coefficient (M = 1.56 × 10^5^ M^−1^·cm^−1^) was used to calculate the amount of MDA. The results were expressed as percentage of control.

### 4.4. Measurement of the Level of Sulfhydryl Groups

The level of sulfhydryl (-SH) groups was determined by the method of Sedlak and Lindsay [46]. Briefly, samples of brain homogenates were mixed with 0.2 M Tris buffer, pH 8.2 and 0.1 M dithionitrobenzoic acid (DTNB) to determine total -SH groups. Non-protein SH groups that reflect the content of non-enzymatic antioxidant defense system (glutathione) were estimated after the addition of 50% TCA to each sample. The tubes were centrifuged at 3000× *g* for 10 min. The absorbance of the supernatants was read within 5 min at 412 nm after the addition of 0.4 M Tris buffer (pH = 8.9) and 0.1 M DTNB against a reagent blank. The content of protein-bound SH groups that reflect the status of protein oxidation during OS was calculated as a difference between total and non-protein SH groups.

### 4.5. Western Blot Analysis

Protein concentrations in the probes were determined according to the method of Lowry [47], using bovine albumin as a standard. Samples containing 50 μg of protein were subjected to SDS-polyacrylamide gel (10%) electrophoresis (Laemmli, 1970, [48]). Samples transferred onto nitrocellulose membranes were incubated overnight (4 °C) with primary monoclonal antibodies anti-SOD1 (1:1000) and anti-SOD2 (1:2000) (Santa Cruz Biotechnology, Dallas, TX, USA) and then with the secondary antibody conjugated with HRP (1:10,000) (Sigma-Aldrich, St. Louis, MO, USA). Polyclonal anti-β actin antibody (Sigma-Aldrich, St. Louis, MO, USA, 1:500) was used as an internal standard. Bands were visualized using the chemiluminescence ECL kit (Amersham, Buckinghamshire, UK), exposed to Hyperfilm ECL, and quantified by densitometric analysis (Image Scanner III (GE Healthcare, LabScan 6.0 Freiburg, Germany) with the Image Quant TL v. 2005 program).

### 4.6. Determination of SOD’s Expression by Quantitative Real Time PCR

Total RNA was extracted from the brain cortex of all experimental groups of animals. Isolation was performed using TRI Reagent (Sigma, St. Louis, MO, USA) according to the method of Chomczyński [49]. RNA (2 µg) was reverse transcribed using random primers and AMV reverse transcriptase (Life Technologies, Forest City, CA, USA). The RT-PCR conditions included reverse transcription at 42 °C for 45 min followed by denaturation at 94 °C for 30 s. TaqMan assays were used for quantitative real time PCR analysis. Rat-specific primers for *SOD1 Rn00566938_m1* and *SOD2 Rn00690588_m1* were used. The probes were obtained from Life Technologies (Forest City, CA, USA). The levels of SODs and actin mRNAs were determined using the TaqMan assay reagents (Life Technologies, Forest City, CA, USA). Quantitative real time PCR (qPCR) analysis was conducted on a Roche LightCycler ^®^ 96 system using 5 μL of RT product, TaqMan PCR Master Mix primers, and a TaqMan probe at a total volume of 20 μL. Cycle conditions for the qPCR were as follows: initial denaturation at 95 °C for 10 min, 45 cycles of 95 °C for 15 s, and 60 °C for 1 min. Each sample was analyzed in triplicate. The relative expression of SODs’ mRNA was normalized to actin (Actb) as a reference gene and calculated on the basis of the _ΔΔ_Ct method.

### 4.7. Measurement of SOD’s Activity

To obtain homogenates for the measurement of SOD activity, the forebrains were homogenized in cold 20 mM HEPES buffer (pH 7.4) containing 1 mM EGTA and 70 mM sucrose. The homogenates were centrifuged at 10,000× *g* at 4 °C and the supernatants were stored on ice and assayed immediately for SOD activity. The activity of the enzyme was determined with a commercially available assay kit (catalog nr 19160, Sigma St. Louis, MO, USA) according to the manufacturer’s instructions. The assay is based on a colorimetric method with a tetrazolium salt that is reduced by superoxide anions (O_2_^−^) to form a formazan dye. SOD activity is determined indirectly as an inhibition of the production rate of O_2_^−^ by xanthine oxidase at 37 °C by measuring the decrease in absorbance of light at 440 nm. SOD activity was measured against standards, calculated using a standard curve and expressed as units (U)/mg protein.

### 4.8. Statistical Analysis

The results are expressed as percentages of the control or as the mean ±SD from 3–4 experiments as identified in the legends of the respective figures. Statistical significance was assessed by Student’s t-test or a one-way-ANOVA with Bonferroni’s or Tukey’s multiple comparison tests to identify the changes that were significantly different from the values of the control or untreated EAE rats using GraphPad PRISM software, version 6.0 (San Diego, CA, USA).

## Figures and Tables

**Table 1:** Changes in the body weight of experimental rats in different phases of EAE.

Body Weight (g)			
Days Post Immunization (d.p.i.)	Control	EAE	EAE + Memantine
0	194.0 ± 8.7	193.6 ± 10.4	195.9 ± 11.8
4	198.4 ± 10.8	190.4 ± 10.1	191.6 ± 9.1
12	210.7 ± 4.3	157.9 ± 9.9 ***	166.0 ± 10.3 ***
20	212.0 ± 3.3	163.2 ± 7.7 ***	182.1 ± 5.1 ***^,###^
25	211.8 ± 2.6	172.0 ± 12.9 ***	188.4 ± 8.8 **^,#^

The values represent the means ± SD from *n* = 64 animals.** *p* < 0.01, *** *p* < 0.001 significantly different vs. control. ^#^ *p* < 0.05, ^###^ *p* < 0.01 vs. experimental autoimmune encephalomyelitis (EAE) rats not subjected to therapy (one-way ANOVA with post hoc Tukey’s test).

**Table 2:** Characteristics of the EAE model and the clinical parameters of EAE rats prior to and after treatment with memantine.

Characteristics/Clinical Parameters	EAE	EAE + Memantine
Animals with clinical symptoms (%)	100	98.44
Animals with severe EAE (%)	76.67	61.11
Lethality (%)	3.33	2.22
Inductive phase (days)	9.5 ± 1.4	11.3 ± 0.8 ***
Cumulative CI (score)	16.93 ± 1.16	9.57 ± 0.67 ***
Duration of disease (days)	17.5 ± 1.4	15.2 ± 1.0 ***
Number of animals	90	90

Administration of the NMDAR antagonist memantine reduced the neurological deficits and improved the condition of the experimental rats during the course of the disease. (CI, cumulative index). The values represent the means ±SD from *n* = 64 animals. *** *p* < 0.001 significantly different vs. experimental autoimmune encephalomyelitis (EAE) untreated rats (Student’s *t*-test).

## Data Availability

The data presented in this study are available at the Laboratory of Pathoneurochemistry, Department of Neurochemistry; Mossakowski Medical Research Institute, Polish Academy of Sciences.
